# Variability in multiple paternity rates for grey reef sharks (*Carcharhinus amblyrhynchos*) and scalloped hammerheads (*Sphyrna lewini*)

**DOI:** 10.1038/s41598-017-01416-w

**Published:** 2017-05-08

**Authors:** M. E. Green, S. A. Appleyard, W. White, S. Tracey, J. Ovenden

**Affiliations:** 1 CSIRO Oceans & Atmosphere, Castray Esplanade, Battery Point, Hobart TAS 7004 Australia; 2 CSIRO Australian National Fish Collection, National Research Collections Australia, Castray Esplanade, Hobart TAS 7004 Australia; 3 Institute for Marine and Antarctic Studies, University of Tasmania, Private Bag 49, Hobart, TAS 7001 Australia; 4 School of Biomedical Sciences, University of Queensland, Chancellors Pl, St. Lucia, Brisbane OLD 4072 Australia

## Abstract

This study assessed the presence and prevalence of multiple paternity (MP) in litters of grey reef sharks (*Carcharhinus amblyrhynchos*) and scalloped hammerheads (*Sphyrna lewini*) opportunistically caught in Papua New Guinea (PNG). Litter size between species were significantly different with an average of 3.3 pups for grey reef sharks and 17.2 pups for scalloped hammerhead. Using 14 and 10 microsatellite loci respectively, we identified MP in 66% of grey reef sharks (4 out of 6 litters) and 100% MP in scalloped hammerheads (5 litters). We found high paternal skew (the uneven contribution of sires per litter) and a positive correlation between female adult size and litter size in scalloped hammerheads but not in grey reef sharks. Differences in the frequency of MP between species and the identification of paternal skew may be linked with mating strategies and post-copulatory mechanisms. Multiple paternity is thought to benefit populations by enhancing genetic diversity therefore increasing the population’s genetic resilience to extrinsic pressures. The identification of MP in two shark species reported here, further elucidates the complex breeding strategies elasmobranchs undertake.

## Introduction

Increasing resolution of molecular tools allows for a greater understanding of shark and ray (elasmobranch) reproductive systems which are often difficult to observe in the wild^1–3^. Elasmobranchs exhibit a variety of reproductive modes including live-bearing (viviparity), egg laying (oviparity)^4^ and parthenogenesis^5^ and also display monogamous and polyandrous mating behaviours^6, 7^. Elasmobranchs do not often form pairs before and/or after mating and do not provide postnatal care to offspring^8^, making their propensity for behavioural monogamy generally low. Instead, it is more likely for females to display polyandrous behaviour, mating with a number of males^8, 9^, the outcome of which may be a single litter, sired by many males and composed of full and half-siblings (sibs) (i.e. multiple paternity)^10^. Polyandry with multiple paternity has a number of benefits^3, 11–13^. Firstly the fitness of the mother is increased as she is more likely to produce offspring; secondly, the adaptive fitness of individuals within litter may be improved as genetic variation is more likely to increase; thirdly, increases in genetic diversity can counteract issues of inbreeding facilitated by close-kin mating (especially for small populations); and finally, multiple paternity can increase the effective population size by providing an opportunity for a greater number of males to mate with an increased number of females^3^.

The occurrence and prevalence of multiple paternity within an elasmobranch litter varies between species, populations and even individuals, but reasons for this are poorly understood^3, 14^. Previous studies have suggested the likelihood of genetic monogamy or polyandry within a litter is dependent on a number of factors including the mother’s size, home range or philopatric tendencies, population size, species-specific behaviours and the presence of post copulatory mechanisms (e.g. sperm storage)^3, 12, 14–19^.

Sharks have life-history characteristics that make them highly susceptible to population declines, e.g. slow growth, delayed maturation and low fecundity^20, 21^. An estimated 25% of all shark and ray species are threatened under the criteria of the International Union for Conservation of Nature (IUCN) Red List, with overfishing considered one of the main causes^21^.

In Papua New Guinea (PNG), grey reef sharks (*Carcharhinus amblyrhynchos*) and scalloped hammerheads (*Sphyrna lewini*) are commonly caught by coastal artisanal and commercial fisheries. Regionally, the level of exploitation of both species is undocumented, making it difficult to assess the status of local populations. Globally, overexploitation has led to international conservation measures for scalloped hammerheads (i.e. listed as Endangered on the IUCN Red List^22^ and included in Appendix II of the Conservation on International Trade in Endangered Species), while grey reef sharks are recognised as Near Threatened (IUCN Red List), thereby demonstrating the capacity to recover if managed accordingly^23^.

Grey reef sharks and scalloped hammerheads differ ecologically; while both species have overlapping distributions, their habitat usage differs. Grey reef sharks have a strong affiliation with reef systems and often smaller individuals will show signs of site attachment to specific reefs^24^. Furthermore, telemetry studies have identified sex-specific movement traits for grey reef sharks, with males more likely to travel to neighbouring reefs than females^25^. Scalloped hammerheads display more complex habitat usage patterns including large ontogenetic differences and broader sex-specific movement traits^26, 27^. Generally, juvenile scalloped hammerheads are found in shallower inshore waters, while adults migrate to deeper continental shelf environments^27^. Genetic analyses suggests females are more likely to display philopatric tendencies, adhering to coastal habitats, while males are known to disperse across oceans^19^. Both grey reef sharks and scalloped hammerheads form large female aggregations^27, 28^ and, once gravid, they are known to move inshore seeking refuge in nursery areas for birthing^19, 25^. Additionally, scalloped hammerheads have post-copulatory mechanisms allowing for long-term (months to years)^29^ sperm storage.

Obtaining mother and litter information for sharks is challenging given mothers are required to be sacrificed for collection of pups, and the common opportunistic nature of sampling regimes often means sample sizes are limited^3, 30^. Recently, MP analyses were undertaken for scalloped hammerheads in southern Africa^30^. Using up to six microsatellite loci, Rossouw *et al*.^30^ identified MP in 46% of 13 litters tested. Given maternal population differentiation has been identified between the regions and differences in average litter sizes, South Africa n = 30^31^ and Indo-Pacific n = 25^32^, it is of interest if rates of MP also differ between regions. Conversely, there has been no assessment of multiple paternity in grey reef sharks from any location. Here we investigated MP in grey reef sharks and scalloped hammerheads captured in the Indo-Pacific Ocean. Given that all studies which have undertaken paternity tests on shark litters have uncovered MP (see review in Rossouw *et al*.^30^) we predict MP will also be found for both species in this current study. However rates of MP are likely to differ given the variation in behaviour, ecology and physiology between the species. Using suites of microsatellite markers, litters were genetically determined as consisting of full or half sibs with an estimate of the number of fathers and their contribution to the litters in each species also obtained. This is the first study to investigate multiple paternity in grey reef sharks and the first for scalloped hammerheads in the Indo-Pacific Ocean.

## Methods

### Sampling and Microsatellite Analyses

**Figure 1:**
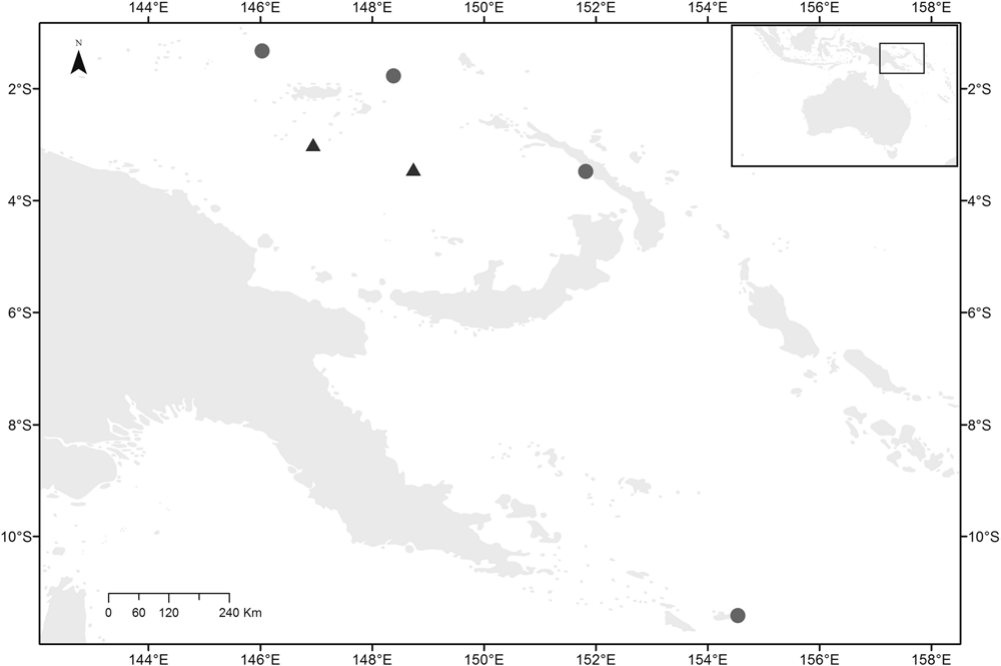
Sample locations for grey reef sharks *C*. *amblyrhynchos* (circles) and scalloped hammerhead *S*. *lewini* (triangles) in Papua New Guinea. Map created using ArcMap 10.2.1 (http://desktop.arcgis.com/en/arcmap/).

Sample collection was undertaken on board commercial fishing vessels operating in PNG between 3^rd^ May 2014 and 6^th^ June 2014. Sampling was undertaken by observers deployed as part of an Australian Centre for International Agricultural Research project led by the National Fisheries Authority (NFA) of PNG and CSIRO to assess shark and ray catches throughout the commercial and artisanal fisheries in PNG (experiments approved by ACIAR and CSIRO; project FIS/2012/102). All samples were collected within a single month from the Bismarck and Solomon Seas (Fig. 1). Tissue samples including fin clips, vertebral chord or muscle were collected from pregnant females and all pups. Observers recorded total length of the adult females and measurements from the smallest and largest pups within a litter.

DNA was extracted using the Wizard^©^ SV Genomic DNA Purification system (Promega, Australia); tissue extractions were undertaken using SV minicolumns following modifications to the manufacturer’s instructions (i.e. overnight tissue digestion; amount of supernatant used to elute DNA was reduced; DNA elution times increased). DNA was quantified using a Nanodrop 8000 UV-Vis Spectrophotometer (Thermo Scientific, USA) and standardised to 20ng/uL.

Microsatellites from pups in each litter were amplified by Polymerase Chain Reaction (PCR) and compared to genotypes in the corresponding mother. Species-specific microsatellite primers for grey reef sharks and scalloped hammerheads were from Momigliano *et al*.^33^ and Nance *et al*.^34^ respectively^33, 34^. In the current study, microsatellite multiplexes were developed to enable cost effective screening. Forward primers were labelled with 6-FAM, VIC, NED and PET proprietary dyes and multiplexed (Table 1). PCR reactions consisted of GoTaq^©^ Colourless Master Mix (Promega, USA), Bovine Serum Albumin (Promega, USA), 0.2 µM of each individual F and R primer (see Table 1 for multiplexes), and 0.8 ng/µl DNA in a 25 µL reaction. For scalloped hammerheads, thermal cycling consisted of initial denaturation at 94 °C × 3 minutes, 35 cycles of 94 °C × 1 minute, 58 °C × 30 seconds, 72 °C × 1 minute and a final extension of 72 °C × 10 minutes. Thermal cycling for grey reef sharks consisted of a touch-down protocol including initial denaturation at 94 °C × 3 minutes, 35 cycles of 94 °C × 1 minute, 5 cycles of 56 °C × 30 seconds, 5 cycles of 54 °C × 30 seconds, 25 cycles of 52 °C × 30 seconds, 35 cycles of 72 °C × 1 minute and a final extension of 72 °C × 10 minutes. Following PCR amplification, Gene Scan^TM^ LIZ 500® size standard (Thermofisher Scientific, USA) and formamide were added to 3 µL of each PCR reaction and 20 µL sample volumes were run on an ABI 3130XL AutoDNA sequencer (Thermofisher, USA). Genotypes were scored and checked by eye using Geneious© R8.1.4 Microsatellite plug-in program (Biomatters Ltd Auckland, New Zealand).

**Table 1:** Characterisation of microsatellite loci for grey reef sharks (*C. amblyrhynchos*) and scalloped hammerheads (*S. lewini*).

Locus Name	n	N_a_	H_o_	H_e_	PIC
C. amblyrhynchos	26				
C. amb11^1^		14	0.938	0.895	0.878
C. amb3^1^		26	0.844	0.921	0.908
C. amb7^1^		8	0.703	0.759	0.715
C. amb2^1^		13	0.887	0.883	0.863
C. amb27^2^		10	0.797	0.823	0.793
C. amb9^2^		6	0.641	0.601	0.530
C. amb28^2^		12	0.844	0.807	0.779
C. amb4^2^		16	0.828	0.81	0.782
C. amb18^3^		25	0.938	0.952	0.942
C. amb15^3^		15	0.746	0.865	0.842
C. amb5^3^		9	0.813	0.766	0.726
C. amb22^3^		4	0.094	0.134	0.129
C. amb25^4^		10	0.906	0.826	0.797
C. amb20^4^		14	0.828	0.883	0.863
*S*. *lewini*	91				
SLE027^1^		9	0.867	0.804	0.773
SLE018^1^		4	0.545	0.516	0.472
SLE089^1^		18	0.966	0.91	0.898
SLE038^2^		7	0.943	0.781	0.744
SLE045^2^		4	0.818	0.721	0.665
SLE054^2^		5	0.685	0.664	0.621
SLE053^3^		12	0.667	0.84	0.817
SLE081^3^		8	0.922	0.787	0.753
SLE071^3^		11	0.582	0.738	0.713
SLE077^3^		13	0.681	0.889	0.873

Number of individual mothers and pups (n), number of alleles (N_A_), observed heterozygosity (H_o_), expected heterozygosity (H_e_) and Polymorphic Information Criteria (PIC).

### Statistical Analysis

For each microsatellite locus, numbers of alleles, allele frequencies, and observed (H_o_) and expected heterozygosities (H_e_) were determined using Genepop web service v4.0.10^35^. Significance of H_o_ and H_e_ tests were estimated by the Markov Chain method including 10,000 dememorizations, 500 batches and 10,000 iterations (not reported). Polymorphic information content (PIC) was estimated using Cervus v3.0^36^.

Analysis of paternity was initially checked by visual inspection of multi-locus genotypes. Secondly, putative fathers (number of sires) and paternal skew within litters were inferred using two programs: Gerud v2.0^37^ which identifies the minimum number of fathers through exclusion calculations, and Colony v2.0.4.5^38^ which uses a maximum likelihood approach. Polygamous mating systems were assumed for both sexes to allow for the assignment of full and half-sibs in Colony. Probability of detecting multiple paternity was calculated post-hoc using PrDM software^39^ (available at http://publish.uwo.ca/~bneff/software.html). Six different scenarios were tested and defined according to the number of pups per litter and the minimum number of fathers identified in Gerud v2.0^37^. These scenarios were defined according to the number of pups observed in the present study (for each species) and the degree of paternity tested in other shark PrDM MP analyses^3, 13, 15^.

## Results

**Figure 2:**
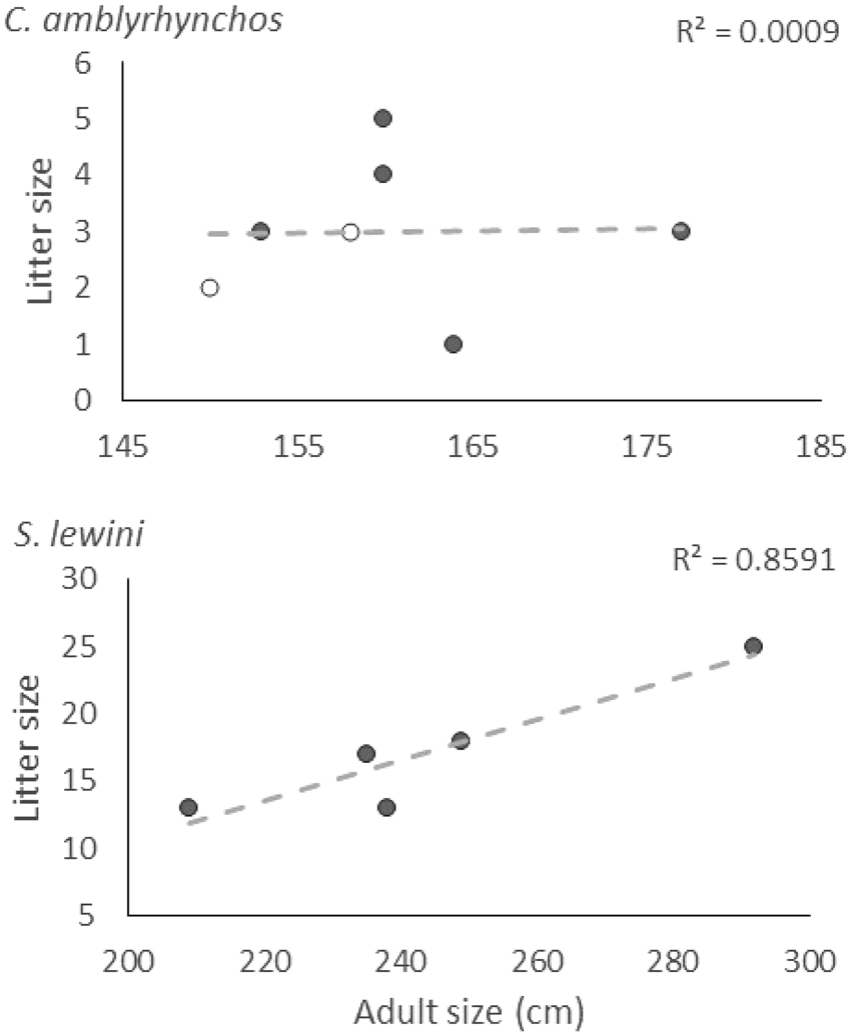
Correlation between adult female length (TL) and litter size for grey reef sharks (*C*. *amblyrhynchos*) and scalloped hammerhead (*S*. *lewini*). Shaded points indicate litter with multiple paternity, unshaded represents litters without multiple paternity.

Six litters of grey reef sharks and five litters of scalloped hammerheads were used to investigate the presence of multiple paternity for sharks captured in PNG waters. Litter size between the species was significantly different (*P* = 0.007, Wilcoxon rank sum test), with grey reef sharks having an average litter of 3.3 pups and scalloped hammerheads an average of 17.2 (Table 2). Sex ratios within litters showed no significant bias towards either sex (*P* > 0.05, chi-square test). Litter size was positively correlated with adult female length for scalloped hammerheads (*P* = 0.023, R^2 ^ = 0.859, Pearson’s rank correlation) but not for grey reefs (*P* = 0.675, R^2^ = 0.000) (Fig. 2). We note however, that these analyses are based on small sample sizes (i.e. litter numbers per species) and should be treated with caution.

**Table 2:** Summary of analysed litters, including female total length, litter size, sex ratio of pups (M:F Ratio), size range of pups, number of sires as estimated by Gerud and Colony, skew (paternal) for grey reef sharks (*C. amblyrhynchos*) and scalloped hammerhead (*S. lewini*).

Species	Total Length (cm)	Litter Size	M:F Ratio	Size range of pups (cm)	# Sires (Gerud)	Skew (Gerud)	# Sires (Colony)
*C*. *amblyrhynchos*	160	4	3:1	51–54	2	2:2	2
*C*. *amblyrhynchos*	160	5	3:2	52–56	2	3:2	3
*C*. *amblyrhynchos*	153	3	0:3	40–41	2	2:1	2
*C*. *amblyrhynchos*	158	3	1:2	54–56	1	-	1
*C*. *amblyrhynchos*	150	2	1:1	45–62	1	-	1
*C*. *amblyrhynchos*	177	3	3:0	20–21	2	2:1	2
*S*. *lewini*	249	18	8:10	46–50	3	6:10:2	8
*S*. *lewini*	292	25	17:8	44–51	3	5:17:3*	7
*S*. *lewini*	238	13	NA	5–7	4	3:5:3:2	4
*S*. *lewini*	209	13	4:9	38–41	2	10:3*	2
*S*. *lewini*	235	17	9:8	42–48	4	8:3:4:2	3

NA Indicates pups were too young to identify sex, *P < 0.05 chi-square test.

Alleles were amplified in microsatellite suites of 14 and 10 loci for all mothers and pups across 26 grey reef sharks and 91 scalloped hammerheads, respectively (Table 1). H_o_ ranged from 0.094–0.938 in grey reef sharks and 0.545–0.966 in scalloped hammerheads. Polymorphic Information Content (PIC) values were generally high, with 86% and 70% of grey reef shark and scalloped hammerhead loci greater than 0.7 respectively. The probability of detecting multiple paternity (PrDM) was highest for scalloped hammerheads (0.94-1), while probabilities were varied and reduced for grey reef sharks (0.47-1; Table 3). Number of loci had less effect than the number of pups within a litter in the detection of multiple paternity. Multiple paternity was identified in 66% of grey reef shark litters (4 out of 6) and in all scalloped hammerhead litters (all five) (Table 2). The number of putative fathers ranged from 1–3 for grey reef sharks and 2–8 for scalloped hammerheads based on Gerud and Colony estimates. In most cases, Colony analysis detected the same or a higher number of sires than Gerud. Paternal skew was identified in two scalloped hammerhead litters indicating an uneven contribution of pups per sire (Table 2).

**Table 3:** Probability to detect multiple males (PrDM) using different suites of microsatellite markers: 14 loci for *C. amblyrhynchos* and 10 loci for *S. lewini* under a number of paternal skew scenarios.

Paternal skew	Litter Size						
	*C*. *amblyrhynchos*			*S*. *lewini*			
	3	4	5	13	17	18	25
2 males (50:50)	0.74	0.88	0.94	1.00	1.00	1.00	1.00
2 males (66.7:33.3)	0.71	0.84	0.91	1.00	1.00	1.00	1.00
2 males (80:20)	0.47	0.59	0.68	0.94	0.98	0.98	1.00
3 males (33.3:33.3:33.4)	0.88	0.96	0.99	1.00	1.00	1.00	1.00
3 males (57:28.5:14.5)	0.78	0.89	0.94	1.00	1.00	1.00	1.00
4 males (25:25:25:25)	0.93	0.99	1.00	1.00	1.00	1.00	1.00

## Discussion

Results from this study provide the first evidence of multiple paternity in grey reef sharks, and the presence of MP in all studied litters of scalloped hammerheads in the Indo-Pacific Ocean. This is the first identification of 100% MP for a species of shark (albeit with a limited number of litters, *n* = 5) and the second within all elasmobranchs studied; 100% multiple paternity (n = 4) has previously been identified in the thornback ray *Raja clavata* ^40^. Multiple paternity was observed in 66% of grey reef shark litters, but the power to detect multiple paternity decreases with decreasing litter size, as shown in PrDM analyses (Table 3). Given the small litter sizes, it is possible analyses presented here underestimate levels of MP for grey reef sharks. Alternatively, we believe small litter sizes may simply create a limited number of embryos available for fertilization by multiple males.

The percentage of litters reported to have MP for grey reef sharks (66%) is comparable to that of other large live bearing sharks, including the sandbar shark *Carcharhinus plumbeus* (40%)^16^. The benefits of polyandrous behaviour have been previously described and include ensuring successful fertility, increasing genetic diversity and genetic fitness (of mother and pups), and reducing close-kin mating (important, if populations are small or inbred)^41–43^. Our observation that polyandrous mating was detected in the larger of the grey reef shark females may have implications for populations exploited in PNG waters. For example, the gear used in longline fisheries including bait and hook size affect the size selectivity of a harvest^44^. If larger individuals are targeted and these individuals are more likely to undertake MP, their removal could mean reduced effective population size and a potential loss in genetic fitness for the population. It is therefore important future work (including larger sample sizes, than this current study) is undertaken to understand the relationship between MP and female size for grey reef sharks.

The finding of 100% multiple paternity in scalloped hammerhead litters in this study contrasts with another study which identified only 46% multiple paternity across 13 litters in South Africa^30^. Interestingly, however, Rossouw *et al*.^30^ reported an average litter size of seven pups, well below the documented litter size for scalloped hammerheads in South Africa (*n* = 30)^31^. Sharks in the Rossouw *et al*.^30^ study were captured in bather protection nets, and it is possible the mothers may have aborted the majority of pups prior to landing, potentially limiting the study to a subset of all pups in the litter. This could lead to an underestimate of the level of multiple paternity for scalloped hammerheads in South Africa.

Multiple paternity is thought to be more common in species that display high levels of philopatry and low dispersal rates, as such behaviour is likely to reduce the chance of individuals breeding with a genetically incompatible (related) partner, thereby decreasing the chance of localized inbreeding depression^3, 11, 43^. For both scalloped hammerhead and grey reef sharks, genetic^19, 45^ and telemetry studies^25, 46^ have revealed strong patterns of female mediated site fidelity and male-biased dispersal. Male dispersal has been prevalent enough to facilitate connectivity (gene flow) between reefs spanning 1,200 km for grey reef sharks^45^ and across ocean basins for scalloped hammerheads^19^. For both species in PNG, it would seem the presence of MP is unlikely to be driven by the threat of close-kin mating or inbreeding depression, given the significant gene flow facilitated by male dispersal in these species shown elsewhere.

Two of the five scalloped hammerhead litters were identified as having significant paternal skews. The presence of paternal skew, (i.e. the uneven contribution of sires to a litter) is thought to be attributed to a combination of female choice, the timing/order of males mating, and sperm competition^18, 30^. The processes of post-copulatory mechanisms are thought to increase the level of paternal skew within a litter^47–50^. Scalloped hammerheads have complex oviducal glands capable of stimulating bundles of sperm to be released, giving control over sperm utilization and its contribution to paternal skew within a litter^29, 51, 52^. Additionally, it is thought that polyandrous mating may create an internal environment within a female that promotes sperm competition, leading to increased fertilization and consequently increased fitness of young (‘sexy-sperm hypothesis’)^53, 54^. This hypothesis suggests females mate with different males to create conditions selecting for the most competitive sperm; which results in male offspring possessing the gene for heightened sperm competitiveness and therefore increasing offspring fitness^54^. It is possible males with heightened sperm competitiveness would sire more pups within a litter creating paternal skew. The mechanisms behind paternal skew in scalloped hammerheads could be one or a combination of factors described here and remains unresolved. The observed lack of paternal skew in grey reef sharks may be connected to the smaller litter size of the species; more litters are required to conclusively verify this hypothesis.

The results of this research concur with similar studies and reiterate the prevalence of MP in sharks. Our results highlight the difference in litter size between the grey reef sharks and scalloped hammerheads and demonstrates differences in levels of multiple paternity. Additionally, the discovery of positive correlations between adult size, litter size and MP suggests genetic mating systems in sharks are complex and may be species- and location-specific. Sample sizes presented here are relatively small and further investigation is required to conclusively understand the relationship between adult size and breeding behaviours. However, a number of studies assessing multiple paternity in sharks (and elasmobranchs more widely) have tested five or less litters^1, 13, 40, 42, 55, 56^ and given the opportunistic nature and difficulties associated with sampling gravid elasmobranchs, the findings from this research provide valuable insight for these two species.
